# Frying stability of sunflower oil blended with jujube (*Ziziphus mauritiana* Lam.) leaf extract

**DOI:** 10.1002/fsn3.247

**Published:** 2015-05-19

**Authors:** Mojtaba Delfanian, Reza Esmaeilzadeh Kenari, Mohammad Ali Sahari

**Affiliations:** ^1^ Graduated from Faculty of Food Science and TechnologyGorgan University of Agricultural Sciences and Natural ResourcesGorganIran; ^2^ Department of Food Science and TechnologySari Agriculture and Natural Resources UniversitySariMazandaranIran; ^3^ Department of Food Science and TechnologyFaculty of AgricultureTarbiat Modares UniversityTehranIran

**Keywords:** Antioxidant, extraction, microwave, ultrasound, *Ziziphus mauritiana*

## Abstract

The aim of present study was to compare the effects of ultrasound‐assisted and microwave‐assisted extraction with solvent extraction method on antioxidant activities of jujube (*Ziziphus mauritiana* Lam.) leaf extracts in stability of sunflower oil during deep frying. The antioxidant activities of the extracts were evaluated by using 2, 2‐diphenyl‐1‐picrylhydrazyl (DPPH˙) radical scavenging and *β*‐carotene bleaching assays. Ultrasound‐assisted extraction was the most effective method on antioxidant activities of extracts and extraction yield of phenolic compounds compared to other extraction techniques. Protective effect of methanol–water extract of jujube leaf obtained with ultrasound‐assisted extraction (ULMW) at 500 and 700 ppm in stability of sunflower oil was compared to synthetic antioxidants by measuring total polar compounds (TPC), carbonyl value (CV), peroxide value (PV), free fatty acids (FFA), oxidative stability index (OSI), conjugated dienes (CD), and trienes values (CT). Results showed ULMW at 700 ppm had higher stabilization efficiency than synthetic antioxidants.

## Introduction

Frying of foods is a very popular way of cooking at home and in fast‐food restaurants (Casal et al. 2010). It is a fast, convenient, and an energy‐efficient cooking method that increases palatability due to fat absorption, crust formation, and pleasant flavors and odors (Yoon et al. 2000). However, it is well known that frying oils used continuously at high temperatures in the presence of oxygen and water from the food being fried are subject to thermal oxidation, polymerization, and hydrolysis, and the resultant decomposition products adversely affect flavor and color (Nor et al. 2008). In addition, undesirable constituents produced from degraded frying oils may even be harmful to health.

Volatile and nonvolatile compounds are formed in vegetable oils during deep frying. Volatile compounds are removed from oil and nonvolatile compounds accumulate in the oil. Nonvolatile compounds are produced primarily by thermal oxidation and polymerization of unsaturated fatty acids (Aladedunye and Przybylski 2013). Nonvolatile compounds are often polar and of high‐molecular weight which have toxic effects on humans and animals (Abdulkarim et al. 2007).

Therefore, synthetic antioxidants such as butylated hydroxytoluene (BHT), butylated hydroxyanisole (BHA), and tertiary butylhydroquinone (TBHQ) are often used to increase the oxidative stability of oils (Delfanian et al. 2015). BHT and BHA have been reported to have toxic and carcinogenic effects (Rop et al. 2011). However, despite high antioxidant activity of TBHQ, its use has been prohibited in some countries, such as Japan and Canada (Suja et al. 2005). Studies have shown that plants are rich sources of antioxidant compounds such as flavonoids, phenolic compounds, tocopherols, carotenoids, and tannins. So, research on natural antioxidants as alternatives to the synthetics has been increased in recent years.

Jujube (*Ziziphus mauritiana* Lam.) is a member of the Rhamnaceae family. The tree (lotus) grows in arid and semiarid regions (Thanatcha and Pranee 2011). The lotus originated from Central Asia and is widely cultivated in Zimbabwe, Thailand, India, and Malaysia (Nyanga et al. 2007). The height of the lotus is about 10 meters and its leaves are small and heart‐shaped which is painted with three prominent veins. Its fruit is round with dark green skin color in young stage which turns to light green or yellow‐green in the mature stage. The flesh is white to yellow‐white that turns red with skin shrinkage when ripe (Dahiru et al. 2006). This fruit has pharmaceutical properties and is a traditional medicine to help improve blood circulation and nervous system as well as a relief to insomnia. Jujube leaf has good antioxidant properties due to the presence of phenolic, flavonoid, and tocopherol compounds (Thanatcha and Pranee 2011).

Nowadays, various techniques such as ultrasound‐ and microwave‐assisted extraction are used to extract bioactive compounds from plants. Ultrasonic waves with a frequency of more than 20 KHz are in two forms of the probes and bath. Ultrasound has high ability to extract phenolic compounds and that is the reason it is being used frequently (Shirsath et al. 2012). Ultrasound cavitation effect can cause damage to the cell wall and thus improve the extraction yield. Sometimes ultrasound waves may possibly destroy the antioxidant compounds and reduce the extraction yield (Cao et al. 2009). Thus, implementation of this method for any plant material requires further investigation.

Microwave‐assisted extraction (MAE) is a novel technology which has been studied extensively in recent years mainly in order to reduce the extraction time and solvent consumption (Amarni and Kadi 2010). It has been reported that natural antioxidants due to their phenolic structure are more exposed to microwave radiation and many researches have been done in order to extract phenolic compounds such as isoflavones (Terigar et al. 2010). It has been concluded that MAE decreased the extraction time and solvent usage and increased the amount of extracted phenolic compounds. This is due to the polarity of phenolic compounds, higher the polarity of a compound, higher the movement during microwave radiation. This leads to a better extraction of such compounds (Proestos and Komaitis 2008).

The aim of this study was to compare the effects of microwave‐ and ultrasound‐assisted techniques on antioxidant activities of jujube (*Ziziphus mauritiana*) leaf extracts with the solvent extraction method. This study also attempted to determine the frying stability of sunflower oil blends with jujube leaf extracts during deep‐frying.

## Materials and Methods

Fresh leaves of jujube (*Ziziphus mauritiana*) were collected from fields in Bandar Abbas (South of Iran). Refined sunflower oil with no added antioxidant was supplied by Rana (Gorgan, Iran) and was stored at −20°C until analysis. All reagents used in the experiments were of analytical grade and obtained mostly from Sigma Chemical Co. (St. Louis, MO). Also, solvents were purchased from Merck (Darmstadt, Germany).

### Extraction

The jujube leaves were shed dried under room temperature at 30 ± 2°C for 5 days (in the dark). In the solvent extraction method, dried powder of the sample (10 g) were mixed with 100 mL of solvents (methanol–water (1:1), water and methanol). The mixtures were stirred in a shaker at 160 rpm away from light at room temperature for 48 h. After extraction, the extracts were filtered and solvents evaporated using a rotary evaporator (Heidolph, Schwabach, Germany) at 50°C. The concentrated extracts were stored until testing at −20°C (Tachakittirungrod et al. 2007).

A microwave oven (Samsung, model: CF3110N‐5, Seoul, Korea) was modified for oil extraction. The modified MAE system consisted of a volumetric flask (250 mL) coupled with a condenser at the top and a magnetic stirrer beneath. The microwave output was 900 W with 2450 MHz frequency and its inner cavity dimensions were 400 × 300 × 250 mm. For each extraction, 10 g of dried leaves was mixed with 100 mL of solvents (water, methanol–water (1:1), methanol) in a 250 mL volumetric flask and placed in the microwave oven with magnetic stirring, 6 min of irradiation was performed (8 sec power on and 15 sec power off to prevent super‐boiling of the solvent). Thereafter, the mixtures were filtered and solvents removed under reduced pressure at 50°C by means of rotary evaporator (Heidolph, Schwabach, Germany). The concentrated extracts were stored in a freezer (Taghvaei et al. 2014).

The ultrasound‐assisted extraction procedure was used for the extraction of jujube leaves with the same solvents and at the same ratio. The mixture was sonicated for 30 min in an ultrasonic bath (Elma s 30 H model, total Power Consumption: 280W, Heating Power: 200W, operating at 37 kHz frequency and internal dimensions: 198 × 106 × 50 cm). The temperature was controlled and maintained at 35°C by circulating water. The extracts were filtered and the remaining steps were similar to those of the previous method (Albu et al. 2004).

### Total phenolic content

The content of phenolic compounds in the extracts was determined with Folin–Ciocalteu reagent following the colorimetric method adapted by Taghvaei et al. (2014). Measurements were carried out in triplicate and calculations were based on a calibration curve obtained with gallic acid. The contents of total phenols were expressed as mg of gallic acid equivalents per g of dry weight (mg GAE.g^−1^ DW).

### Radical scavenging activity

DPPH (2, 2‐diphenyl‐1‐picrylhydrazyl) radical scavenging activity of extracts was measured according to the method of Xu and Chang (2007) and modified slightly. The DPPH ˙ solution was prepared by dissolving 5.9 mg of DPPH ˙ in ethanol (100 mL). Accurately, 3.8 mL of DPPH ˙ ethanolic solution was added to 0.2 mL of extracts. The mixture was shaken vigorously for 1 min and left to stand at room temperature in the dark for 30 min. Absorbance was measured against the blank reagent at 517 nm. All determinations were carried out in triplicate. The radical scavenging activity was calculated according to Eq. (1) below:

(1) $$\%\text{Inhabition}\;=\;\frac{A0-A1}{A0}\;\times \;100$$

A0 and A1 are the absorbance of blank and sample, respectively.

Lipid peroxidation inhibition activity was determined, using the *β*‐carotene bleaching assay as described by Lagha‐Benamrouche and Madani (2013). About 0.2 mL of extract was added to 5 mL of *β*‐carotene/linoleic acid solution. The absorbance of the samples was measured at 470 nm at initial time (*T* = 0) against a blank, consisting of an emulsion without *β*‐carotene. The remaining samples were placed in the dark for 24 h. Then, the absorbance of each sample was measured at 470 nm. The radical inhibition activity was calculated according to Eq. (2) below:

(2) $$\%\text{Inhibition}\;=\left[1-\frac{{\text{Abss}}^{24}-{\text{Abss}}^{0}}{{\text{Absc}}^{24}-{\text{Absc}}^{0}}\right]\;\times \;100$$

whereas the Abss^24^ is the absorbance of the antioxidant after 24 h, Absc^24^ is the absorbance of control after 24 h, Abss^0^ is the absorbance of the antioxidant at *t* = 0 and Absc^0^ is the absorbance of control at *t* = 0.

### Frying conditions

The jujube leaf extract in levels of 500 and 700 ppm, BHA, BHT, and TBHQ in level of 100 ppm were added to sunflower oil. Sunflower oil without antioxidant addition was used as negative control. Oil sample (2.5 L) was placed in a fryer oven of 2.5 L capacity (Tefal model 1250, France) and heated at 185 ± 5°C for 24 h. A batch of 20 g of potato pieces (7.0 × 0.5 × 0.3 cm) was fried for 7 min. After a time of heating (0, 4, 8, 12, 16, 20, and 24) 20 g of frying oil samples was stored until testing at −20°C (Farhoosh and Moosavi 2008).

### Oil stability analysis

The Total polar compounds was determined according to the method described by Schulte (2004). The carbonyl value (CV) of the oil samples was measured according to the method developed by Endo et al. (2001) using 2‐propanol and 2,4‐decadienal as solvent and standard, respectively. Results were expressed in micromols of 2, 4‐decadienal per gram of oil. Peroxide value (PV) expressed in milliequivalents of active oxygen per kilogram (meq O_2_/kg of oil) was determined according to Shantha and Decker (1994). The contents of conjugated dienes (CD) and trienes (CT) were calculated according to the method described by Urbancˇicˇ et al. (2014), which is based on the measurement of absorbance solution at 234 nm and 270 nm for CD and CT, respectively. The free fatty acids content (FFA) expressed as free oleic acid percentage, was determined by titration of an accurate sample solution, dissolved in ethanol/ether (1:1, v/v), with 0.1 mol/L sodium hydroxide solution, using phenolphthalein as indicator (Farhoosh et al. 2012). For evaluation of oxidative stability index a Metrohm 743 Rancimat instrument (Herisan, Switzerland) was used in the experiment. Air supply was maintained at 15 L/h and the temperature was kept at 110°C (Farhoosh and Tavassoli‐Kafrani 2010).

### Statistical analysis

The data were subjected to analysis of variance (ANOVA) and the significance of the difference between means was determined by Duncan's multiple range test (*P* < 0.05), using SPSS statistical software (version 19; SPSS Inc., Chicago, IL).

## Results and Discussion

Table 1 shows the extraction yield of jujube leaf which range from 9.82 to 15.02%. Results showed that the methanol–water extract (1:1) was the most effective solvent for extraction of bioactive compounds compared to water and methanol. This is in accordance with the findings of Hemwimol et al. (2006). The extraction yields of different techniques in descending order were ultrasound > microwave‐assisted > solvent extraction. This shows that ultrasound‐assisted extraction was the best technique for the extraction of bioactive compounds from the jujube leaf. Our results concurred with previously published results such as the works by Hemwimol et al. (2006) and Rodríguez‐Rojo et al. (2012) that reported the ultrasound‐assisted extraction method was more effective in extraction of plant materials compared to other techniques.

**Table 1:** Extraction yield and total phenolic contents of jujube leaf extracts

Sample	Extraction yield (%)	Total phenolic content (mg/g)
SLM	10.04e	125.63e
SLW	9.82f	119.62f
SLMW	12.53c	152.03d
MLM	11.62d	265.32c
MLW	10.23e	123.75e
MLMW	14.25b	258.33c
ULM	12.56c	314.71b
ULW	10.14e	148.52d
ULMW	15.02a	365.22a

Mean ± SD. Significant differences in a same column are shown by different letters (*P* < 0.05).

Note: Solvent extracts of jujube leaf by methanol (SLM), water (SLW) and Methanol–water (SLMW). Microwave‐assisted extracts of jujube leaf by methanol (MLM), water (MLW) and methanol–water (MLMW). Ultrasound‐assisted extracts of jujube leaf by methanol (ULM), water (ULW) and methanol–water (ULMW).

Polyphenolic compounds are widely distributed in different parts of plants (Katalinic et al. 2013) and reports showed that there is a positive relation between total phenolic content and antioxidant activity in many plant species (Aladedunye and Matthäus 2014). There were significant differences (*P* < 0.05) between phenolic compounds of extraction techniques with different solvents (Table 1). There were various phenolic contents in the jujube leaf extracts ranging from 119.62 to 365.22 mg GAE/g. Jujube leaf extract was previously reported to have total phenolic compounds in the range of 1321.98 mg GAE/100 g (Ikram et al. 2009) to 2352.5 mg GAE/100 g (Lamien‐Meda et al. 2008). The composition of leaves and phenolic content depends on the genetic and environmental factors as well as post‐harvest processing conditions (Milivojevic et al. 2012). Our results indicated that the methanol–water (1:1) had better effects on extraction of phenolic compounds compared to other solvents in the same extraction conditions. Also, we found that ultrasound‐assisted extraction had a greater effect on the extraction of phenolic compounds. Goli et al. (2005) and Yasoubi et al. (2010) reported similar results indicating that the ultrasound‐assisted extraction was the most effective in extraction of phenolic compounds in comparison with microwave‐assisted and solvent extraction techniques.

Table 2 shows the ability of jujube leaf extracts to scavenge DPPH ˙ radical as the inhibition percentage at concentrations of 100 to 700 ppm. In this assay, similar to previously published results such as the works by Chirinos et al. (2011) and Akkol et al. (2012), the results indicated that scavenging‐free radicals increased as concentrations of the extracts increased which due to increasing amounts of phenolic compounds at higher concentrations of the extracts. With increasing concentrations of phenolic compounds, the number of hydroxyl groups available in the reaction medium increased. So, the possibility of hydrogen donation to free radicals increased (Rajaei et al. 2010). The results showed ULMW at concentrations of 100 to 700 ppm had the highest DPPH ˙ radical‐scavenging capacity between other extracts. This was in agreement with González‐Montelongo et al. (2010) who mentioned that ethanol‐water (1:1) extract was the best to scavenge DPPH ˙ free radical. Also, our results indicated that the ultrasound had better performance on antioxidant activity of jujube leaf in scavenging of free radicals. These results concurred with previously published results such as the work by Rodríguez‐Rojo et al. (2012) in which they evaluated effects of different extraction methods on antioxidant activity of rosemary extract.

**Table 2:** Antioxidant activities of jujube leaf extracts at different concentration analyzed by DPPH^•^ and *β*‐carotene assays

Sample	100 ppm	300 ppm	500 ppm	700 ppm
DPPH^•^				
SLM	23.35e	37.42e	43.72e	46.27e
SLW	19.82g	32.66f	41.53f	45.33f
SLMW	25.47d	37.01e	45.65d	48.55d
MLM	25.23d	39.65d	45.72d	46.58e
MLW	22.75f	37.42e	45.23d	46.33e
MLMW	25.51d	42.53b	48.04b	50.02b
ULM	28.45b	40.22c	46.62c	50.27b
ULW	26.32c	42.54b	48.74b	49.66c
ULMW	31.42a	47.44a	50.63a	52.73a
*β*‐carotene				
SLM	30.45f	46.72g	49.03f	50.03e
SLW	32.23e	46.58g	49.23f	50.88e
SLMW	34.52d	49.73e	50.44e	51.23d
MLM	32.56e	48.45f	50.22e	52.12c
MLW	35.66c	50.12d	52.65d	51.31d
MLMW	37.42b	52.65c	53.84c	53.04b
ULM	34.74d	52.33c	52.12d	53.14b
ULW	37.02b	55.02b	53.66b	55.52a
ULMW	38.16a	57.23a	54.28a	55.67a

Mean ± SD. Significant differences in a same column are shown by different letters (*P* < 0.05).

*Note*: Solvent extracts of jujube leaf by methanol (SLM), water (SLW) and Methanol–water (SLMW). Microwave‐assisted extracts of jujube leaf by methanol (MLM), water (MLW) and methanol–water (MLMW). Ultrasound‐assisted extracts of jujube leaf by methanol (ULM), water (ULW) and methanol–water (ULMW).

The antioxidant activities of the jujube leaf extracts were measured by bleaching of *β*‐carotene which is indicated in Table 2. The extracts obtained from different methods and solvents, showed different degrees of antioxidant activity. Methanol–water extracts showed maximum antioxidant activities with significant differences than other solvents. The ULMW indicated better performance in prevention of *β*‐carotene oxidation in all tested concentrations. In general, the solvent extraction method had comparatively a poor effect on the antioxidant activity of jujube leaf, whereas samples extracted by the ultrasound‐assisted extraction technique at different concentrations had the highest inhibitory effects in prevention of *β*‐carotene oxidation. Our results were in agreement with previously published results of Esmaeilzadeh Kenari et al. (2014).

From the evaluation of the antioxidant properties of extracts in vivo, it is realized that ultrasound‐assisted extract of jujube leaf by methanol–water (ULMW) at 500 and 700 ppm had the highest antioxidant activity. Therefore, ULMW was used to assess its effect on stability of sunflower oil during frying process.

### Oil stability analysis

**Figure 1:**
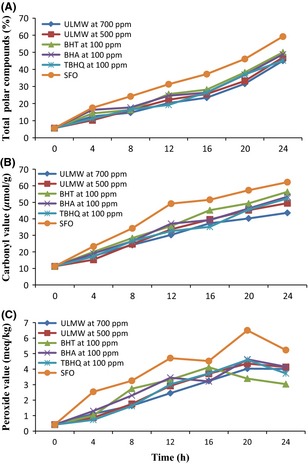
Changes in total polar compounds (A), carbonyl value (B) and peroxide value (C) of sunflower oil during deep frying.*Note*: Ultrasound‐assisted extract of jujube leaf by methanol–water (ULMW), Sunflower oil with no added antioxidant (SFO).

Determination of TPC is one of the most important tests and a valid criterion for assessing the thermal oxidative degradation of the oils during deep frying (Stier 2013). Figure 1 shows changes in TPC content of the oil samples during the deep frying process. In many European countries, the TPC value is considered as a major oil degradation indicator and it is acceptableat maximum 25–27% for used frying oil (Abdulkarim et al. 2007). Fresh sunflower oil had a TPC content of 5.6%, reflecting the good quality of the oil used, as TPC content of unused oils normally ranges between 0.4% and 6.4% (Lumley 1988). The TPC content increased during frying and had high correlation coefficient with frying time. This result concurred with the results of Matthäus (2006), Bansal et al. (2010), and Osawa et al. (2012). The TPC of the oils containing ULMW of 500 and 700 ppm, BHT, BHA, TBHQ, and SFO after 24 h of frying were 47.01, 45.05, 50.01, 48.82, 45.23, and 59.22%, respectively. These results showed that SFO reached 25% TPC (degradation limit for regulation purposes) after 9.4 h of deep‐frying cycles, while the oil with the addition of ULMW 500 and 700 ppm, BHT, BHA, and TBHQ reached 25% TPC after 13.1, 17.2, 12.5, 12.8, and 15.2 h deep frying cycles. Therefore, the thermo‐oxidative stability of sunflower oil increased significantly in the presence of extracts compared to control oil. During frying time observed the rate increase in amount of TPC in 700 ppm of ULMW was relatively slower than that of other oil samples and later reached the critical limit (25%). So that the rate of discard of oil samples was as follows:

$$\begin{array}{c} \text{SFO}\;gt;\;\text{BHT}\;gt;\;\text{BHA}\;gt;\;\text{ULMW}\;500\;\text{ppm}\;gt;\;\text{TBHQ}\;gt;\\ \text{ULMW}\;700\;\text{ppm} \end{array}$$

These results were in agreement with previous studies results such as the works by Casarotti and Jorge (2014) and Urbancˇicˇ et al. (2014) which indicated that rosemary extract was more effective in reducing polar compounds production in frying oil compared to synthetic antioxidants.

Changes in CV of the oil samples during the frying process are shown in Figure 1. Carbonyl value measures secondary products of oxidation such as aldehydes and ketones (Endo et al. 2001). On the basis of the National Standards of Japan, frying oils containing more than 50 *μ*mol/g of CV should be discarded because undesirable changes have occurred in their flavor (Hara et al. 2006). Results showed the CV of a set of oil samples increased and reached a maximum value during frying and then decreased. The result concurred with previously published results of Farhoosh and Kenari (2009) and Farhoosh and Tavassoli‐Kafrani (2010). According to Farhoosh and Kenari (2009) during the prolonged frying period, carbonyl compounds may convert into new compounds that are not measurable with the CV test. There were no significant differences between the initial CV of the oil samples (11.25 *μ*mol/g). The CV for sunflower oil containing 500 and 700 ppm of ULMW was lower than the critical limit (51 *μ*mol/g) during the frying process. Whereas, the oil containing BHT, BHA, and TBHQ after 24 h and SBO after 12 h reached the maximum CV. Therefore, ULMW at 700 ppm showed higher antioxidant activity compared to other oil samples in prevention of carbonyl compounds increase during the frying process.

Figure 1 shows changes in PV of the oil samples during the deep frying process at 180°C. The PV of fresh oil should be <2 meq O_2_/kg (Ramadan et al. 2006). It was generally observed that the extract significantly reduced PV of oils compared to control oil (*P* < 0.05). There was an initial increase in PV of SFO and BHA from 0 to 12 h, for BHT from 0 to 16 h, for TBHQ, ULMW at 500 and 700 ppm from 0 to 20 h and after that the rate slowed down. Peak values for PV were obtained and are as follows:

BHT (4.12 meq O_2_/kg after 16 h), BHA (4.36 meq O_2_/kg after 20 h), ULMW at 500 ppm (4.36 meq O_2_/kg after 20 h), ULMW at 700 ppm (4.03 meq O_2_/kg after 20 h), TBHQ (4.6 meq O_2_/kg after 20 h), and SFO (6.5 meq O_2_/kg after 20 h).

The PV decreased in oil samples after the peak was reached. The PV results were in agreement with previously published results of Casal et al. (2010) and Farhoosh et al. (2012). Rapid rise in PV of soybean oils containing BHT, BHA, TBHQ, and SFO showed that they were more sensitive to oxidation degradation. While, 700 ppm of ULMW indicated greater ability to prevent an increase in PV as compared to the other oil samples. The PV test is not a valid parameter to assess oils' oxidative changes during the deep frying process, because peroxidase under frying conditions are unstable and are converted into other compounds such as carbonyl and aldehyde that cause PV abatement (Sulieman et al. 2006).

**Figure 2:**
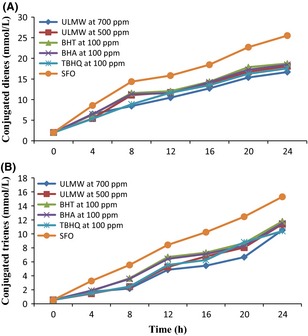
Changes in conjugated dienes (A) and trienes (B) values of sunflower oil during deep frying. *Note*: Ultrasound‐assisted extract of jujube leaf by methanol–water (ULMW), Sunflower oil with no added antioxidant (SFO).

Changes in CD, which represent the degree of production of primary oxidation products, are shown in Figure 2. The CD values of sunflower oil treated with ULMW at 500 and 700 ppm were significantly different from the control oil, and also from the oils with added BHA, BHT, and TBHQ. The CD values in oils containing 500 ppm of ULMW and TBHQ with no significant difference (*P* > 0.05) at 4 h and in oil with 700 ppm of ULMW at other frying times (8, 12, 16, 20, and 24 h) were lower than other oil samples. At the end of the 24 h frying process, the CD values for the sunflower oil containing 500 and 700 ppm of ULMW, BHT, BHA, and TBHQ were 18.08, 16.67, 18.74, 18.45, and 17.54 (mmol/L), respectively.

The CT values of oils treated with antioxidants were significantly different from the control oil (Fig. 2). The CT value of control oil at the end of 24 h of frying was greater than that of oils treated with jujube leaf extracts and synthetic antioxidants. Generally, CT values for sunflower oil containing 500 and 700 ppm of ULMW, BHT, BHA, and TBHQ were 11.4, 10.5, 11.8, 11.5, and 10.4 (mmol/L), respectively. Therefore, the 18.08 at 700 ppm indicated a greater ability to reduce the production of conjugated compounds compared to other oils treated with antioxidants. These results were approved by previous studies of Casarotti and Jorge (2014) and Urbancˇicˇ et al. (2014). In this study, similar to those reported by Chirinos et al. (2011) and Bou et al. (2012), the CD and CT values increased with increasing frying time. Abdulkarim et al. (2007) showed that due to the formation of conjugated dienes and trienes, the absorption increase is proportional to the uptake of oxygen and formation of peroxides during early stages of oxidation. However, the increase in conjugated dienes was considerably higher compared to the conjugated trienes, which will be specifically due to the high content of linoleic acid in sunflower oil.

**Figure 3:**
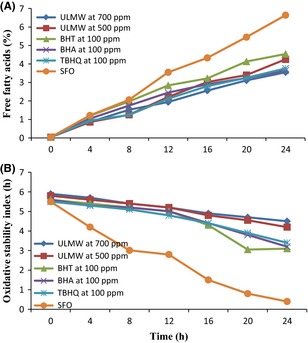
Changes in free fatty acids (A) and oxidative stability index (B) of sunflower oil during deep frying. *Note*: Ultrasound‐assisted extract of jujube leaf by methanol–water (ULMW), Sunflower oil with no added antioxidant (SFO).

Changes in the FFA content of the oil samples are shown in Figure 3. The FFA is used as an indicator for assessment of oil deterioration during frying (Abdulkarim et al. 2007). There was no significant difference between the initial FFA (0 h) of oil samples. However, in all samples, the FFA showed a trend to increase from the beginning of the frying period to the end of the experiment, similar to the results reported by Kim and Choe (2008) and Wang et al. (2012). The FFA content at the end of 24 h of frying for soybean oil containing 500 and 700 ppm of ULMW, BHT, BHA, TBHQ, and SFO reached to 4.24, 3.55, 4.54, 3.65, 3.76, and 6.64%, respectively. However, the values of oil samples treated with natural and synthetic antioxidants were found to be significantly (*P* < 0.05) lower than the control oil. These results indicated that natural antioxidants in ULMW of 700 ppm protected sunflower oil from hydrolysis better than synthetic antioxidants. Results concurred with the results of Casarotti and Jorge (2014) and Urbancˇicˇ et al. (2014) which examined the antioxidant effect of rosemary extract in soybean and sunflower oil.

Changes in the OSI of oils during the deep‐frying process are shown in Figure 3. The Rancimat method is often used for predicting oxidative stabilities of oils under deep frying conditions. The oxidation stability of sunflower oil was greatly improved in the presence of extracts. Similar to results reported by Nor et al. (2008) and Casal et al. (2010), it was observed that stability of oil samples decreased gradually during the frying process. The rate of oxidation of oil samples was as follows:

$$\begin{array}{c} \text{SFO}\;gt;\;\text{BHT}\;gt;\;\text{BHA}\;gt;\;\text{TBHQ}\;gt;\;\text{ULMW}\;500\;\text{ppm}\;gt;\;\\ \text{ULMW}\;700\;\text{ppm} \end{array}$$

Therefore, results indicated that the maximum stability in sunflower oil was obtained by addition of 700 ppm of ULMW. OSI results were in agreement with previously published results such as the work of Taghvaei et al. (2014) and Aladedunye and Matthäus (2014).

## Conclusions

This study showed that the ultrasound‐assisted extraction method had the better effect in antioxidant activity of jujube leaves extracts in the DPPH and β‐carotene assays compared to other techniques. Therefore, it is suggested that the best method for the extraction of phenolic compounds from jujube leaf is the ultrasound‐assisted extraction technique. Results also confirmed that the protection offered by ULMW at 700 ppm is better than that of the widely used synthetic antioxidants such as BHA, BHT, and TBHQ, in terms of total polar compounds, carbonyl value, peroxide value, oxidative stability index, free fatty acids, conjugated dienes, and trienes. In general, jujube leaf extract showed suitable antioxidant capacity in sunflower oil and could be used as a substitute for synthetic antioxidants to increase the shelf life of vegetable oils.

## Conflict of Interest

None declared.
